# Epidemiological Investigation of Allergic Rhinitis in Yulin, Northwest China: A Prospective Case-Control Study

**DOI:** 10.7759/cureus.87384

**Published:** 2025-07-06

**Authors:** Xin Liu, Na Gao, Cairong Bai, Yali Zuo, Mei Ma, Zhenxing Zheng, Wendong Hao

**Affiliations:** 1 Department of Allergy, Yulin Hospital, The First Affiliated Hospital of Xi'an Jiaotong University, Yulin, CHN; 2 Department of Medical Administration, Yulin City Health Commission, Yulin, CHN; 3 Department of Epidemiology and Public Health, Yulin City Center for Disease Control and Prevention, Yulin, CHN

**Keywords:** antibiotic exposure, ar: allergic rhinitis, artemisia desertorum spreng, pollen allergens, risk factors

## Abstract

Background

Allergic rhinitis (AR), a chronic inflammatory condition of the nasal mucosa mediated by immunoglobulin E (IgE) responses to environmental allergens, represents a significant and escalating global public health challenge. Yulin City ("China's Kuwait") borders the ecologically restored, *Artemisia*-dominant Mu Us Desert. An estimated 27% of its population suffers from seasonal AR, yet local epidemiology remains uncharacterized.

Objective

To elucidate region-specific allergens and risk factors for AR in Yulin's unique semi-arid ecosystem.

Methods

In this prospective case-control study conducted across 15 municipal hospitals in Yulin, Northwest China, we recruited 300 patients with physician-diagnosed AR and 300 controls without AR from allergy and otolaryngology clinics between August 1, 2023, and July 31, 2024. Cases and controls were matched on age using individual matching (1:1 ratio) with a caliper of ±5 years. Control participants were systematically screened to exclude AR diagnosis through clinical evaluation and symptom assessment. All participants underwent questionnaire surveys and had peripheral venous blood drawn for complete blood count (CBC), C-reactive protein (CRP), and allergen-specific IgE testing.

Results

This study enrolled 300 AR patients (mean age: 38.17 years; 183 [61.0%] female) and 300 matched controls (mean age: 41.67 years; 194 [64.6%] female). Baseline characteristics, including gender, age, geographic residence, occupation, and prior antibiotic use, demonstrated significant intergroup differences (all *P*<0.01 by chi-square/t-tests). Among AR patients, serum allergen-specific IgE testing identified three predominant allergens: *Artemisia desertorum *Spreng (181 [60.3%]), common ragweed (70 [23.3%]), and pine (62 [20.7%]). Multivariate logistic regression analysis, adjusted for gender, age, residence, and occupation, revealed antibiotic exposure as an independent risk factor for AR (adjusted odds ratio [aOR]=1.70; 95% confidence interval [CI]: 1.22-2.37; *P*<0.01). This association indicates a 70% increase in the odds of developing AR with antibiotic use.

Conclusion

The prevention and control of AR in Yulin should focus on managing *Artemisia desertorum* Spreng allergens and antibiotic use. These findings provide a scientific basis for optimizing the allocation of regional health resources.

## Introduction

Allergic rhinitis (AR), a chronic inflammatory condition of the nasal mucosa mediated by IgE responses to environmental allergens, represents a significant and escalating global public health challenge. Characterized by symptoms such as rhinorrhea, nasal congestion, sneezing, and pruritus, AR substantially diminishes quality of life, disrupts sleep, impairs work productivity and school performance, and imposes considerable economic costs on healthcare systems worldwide [1]. Crucially, AR is a major comorbidity and risk factor for asthma, with epidemiological studies consistently showing that over 80% of asthmatic patients also suffer from AR. This association frequently leads to worsened asthma severity, poorer control, and increased healthcare utilization [1,2]. Consequently, understanding the local epidemiology, identifying the predominant allergens, and elucidating associated risk factors are fundamental prerequisites for developing effective, region-specific prevention and management strategies.

Yulin City, located in the northernmost part of Shaanxi Province, Northwest China, presents a unique and critical setting for investigating AR. Encompassing a vast administrative area of 43,578 km^2^, Yulin is not only geographically significant but also holds immense economic importance [3]. Often dubbed China's "Kuwait" due to its vast energy reserves, primarily coal, it hosts the Shenfu Coalfield, ranked among the world's largest coalfields [4]. This resource wealth drives a local economy, which had a GDP per capita of approximately $32,500 and supported a registered population of 3.85 million people according to the 2020 census.

Adjacent to Yulin lies the expansive Mu Us Desert, a semi-arid region covering approximately 42,200 km². This landscape has undergone a remarkable transformation over recent decades due to systematic, large-scale ecological restoration efforts. Intensive vegetation rehabilitation programs focused on establishing drought-tolerant pioneer species, crucial for dune fixation and microhabitat creation, have successfully stabilized an estimated 80% of the desert area [5]. *Artemisia *species have emerged as the dominant flora within these managed ecosystems, playing a pivotal role in China's northern desertification control strategies [6]. While this greening represents a significant ecological achievement, the proliferation of *Artemisia*, a genus renowned for producing highly allergenic pollen [7], introduces a substantial potential public health concern regarding respiratory allergies.

Despite the unique confluence of factors, large-scale ecological transformation, significant industrial activity, and a sizable population, comprehensive epidemiological data on AR specifically within Yulin City remain strikingly scarce. Symptom exacerbation occurs predominantly in summer and falls for 80-90% of affected individuals, peaking between July and September [8]. Preliminary local public health surveillance estimates indicate a significant public health concern, with approximately 27% of the Yulin population suffering from seasonal AR. This period aligns precisely with the peak pollen season for dominant local plants, particularly *Artemisia* [9]. Furthermore, the region is susceptible to extreme weather events that have demonstrable impacts on respiratory health. A striking example occurred on September 11, 2018, when a major thunderstorm event triggered an overwhelming surge in pediatric emergency and outpatient visits for acute respiratory distress at Yulin Pediatric Hospital [8]. D'Amato et al. documented this event, highlighting the acute vulnerability of the local population, especially children, to environmental triggers capable of exacerbating conditions such as AR and asthma, potentially through mechanisms such as pollen grain rupture and allergen release [10].

The limited existing literature fails to adequately characterize the specific spectrum of major aeroallergens driving AR sensitization and symptoms in Yulin, quantify the precise prevalence and severity across different demographic groups, identify key modifiable environmental and lifestyle risk factors beyond seasonality, or thoroughly investigate the complex interplay between the successful *Artemisia*-dominated ecological restoration, industrial air pollution, climatic factors like thunderstorm activity, and the respiratory allergy burden. This critical lack of localized, granular data significantly hinders the development of targeted public health interventions, evidence-based clinical management guidelines, and effective personalized allergen avoidance advice for Yulin's residents. Therefore, this prospective case-control study aims to directly inform the design and implementation of effective primary and secondary prevention strategies, improve diagnostic accuracy and management protocols within the local healthcare system, and ultimately contribute to alleviating the significant health burden imposed by AR on the population of Yulin City.

## Materials and methods

Study design and setting

In this prospective case-control study conducted across 15 municipal hospitals in Yulin, Northwest China, we recruited 300 patients with physician-diagnosed AR meeting the Allergic Rhinitis and its Impact on Asthma (ARIA) 2025 guidelines [11] and 300 age-matched controls without AR from allergy and otolaryngology clinics between August 1, 2023, and July 31, 2024. The case group and control group were paired in a 1:1 ratio. Controls were matched to cases solely on age (±5 years) using frequency matching. Patients with concurrent allergic diseases, including asthma, atopic dermatitis, urticaria, and allergic skin rashes, were excluded from this study. Control participants were systematically screened to exclude AR diagnosis through clinical evaluation and symptom assessment.

Our sample size of 300 cases and 300 matched controls was determined a priori using power analysis (α=0.05, power=0.90) to detect an expected odds ratio of 1.8 with an exposure prevalence of 35%. This exceeds the minimum requirement of 532 total subjects calculated via PASS software, ensuring robust detection of clinically relevant effects. The institutional review boards of participating hospitals approved the study protocol. Informed consent was obtained from all participants included in this study.

The data collection instrument underwent formal content validation by an expert panel (three allergists, two epidemiologists), achieving a scale-level content validity index (S-CVI) of 0.91 (>0.90 benchmark). In pilot testing with 35 participants (excluded from main analysis), Cronbach's α was 0.87 for symptom domains. Discriminant validity was confirmed by significantly higher scores in AR cases versus controls (24.3±3.1 vs. 8.7±2.4; *P*<0.001, Cohen's d=2.1).

Laboratory investigators were fully blinded to the case/control status throughout testing. All samples were de-identified using coded labels (ID-001 to ID-600), with the key stored independently by the biostatistics team. This ensured objective analysis without knowledge of group assignment.

Epidemiological questionnaire for AR

This study used an epidemiological questionnaire designed to investigate AR, aiming to collect multi-dimensional data on key variables such as age, gender, occupation, educational level, antibiotic use history, and family history of atopic conditions. By integrating these demographic, behavioral, and genetic factors, the questionnaire seeks to identify potential risk factors and assess the prevalence of AR across different populations. The design of the instrument is informed by validated methodologies from prior research, ensuring robustness in data collection and analysis [12,13]. Our study employed two standardized instruments: the Score For Allergic Rhinitis (SFAR) to quantify symptom severity and the Rhinoconjunctivitis Quality of Life Questionnaire (RQLQ) to assess disease-specific quality of life impacts.

Allergen-specific serum IgE testing

Serum-specific IgE against common allergens was quantified using a fluorescent enzyme immunoassay (FEIA) based on the ImmunoCAP technology. Serum samples were collected from participants, centrifuged at 3000×g for 10 minutes to isolate serum, and then diluted 1:10 with sample diluent (Phadia AB, Uppsala, Sweden). Testing was performed on the Phadia 2500 system (Thermo Fisher Scientific, Waltham, MA, USA), a fully automated platform utilizing immobilized allergen extracts conjugated to fluorescently labeled microspheres. Following incubation with diluted serum, bound IgE was detected via anti-human IgE antibodies labeled with beta-galactosidase. Fluorescence intensity, proportional to IgE concentration, was measured by the system's optical module. Results were analyzed using Phadia LabLink software, with reference ranges defined as class 0 (<0.35 kU/L) to class 6 (>100 kU/L). Quality control was ensured using manufacturer-provided calibrators and control sera (levels A-C).

Complete blood count and C-reactive protein tests

In this study, complete blood count (CBC) and C-reactive protein (CRP) were evaluated using standardized laboratory methods. The CBC and CRP tests were systematically performed to characterize inflammatory endotypes in AR. Specifically, eosinophil counts (CBC) quantified Th2-driven inflammation, while CRP levels assessed systemic inflammation linked to the severity of AR. These biomarkers served as predefined secondary endpoints to explore phenotype-endotype correlations, not as diagnostic criteria. For CBC, EDTA-K2 anticoagulated venous blood (2 mL) was analyzed on a Sysmex XN-1000 automated hematology analyzer (Sysmex Corporation, Kobe, Japan), which quantifies parameters including leukocyte differential, hemoglobin, and platelet count. CRP levels were measured via an immunoturbidimetric assay on the same instrument's integrated module, using Sysmex original reagents, following manufacturer protocols. All procedures adhered to clinical laboratory standards for precision and accuracy.

Data analysis

Categorical variables were compared using χ² tests or Fisher exact tests as appropriate. Multivariable logistic regression models adjusted for potential confounders were employed to identify risk factors associated with AR, with statistical significance set at *P*<0.05 (two-tailed).

## Results

From August 1, 2023, to July 31, 2024, this study included 300 patients with AR (mean age 38.17 years; 183 [61.0%] female) and 300 control participants without AR (mean age: 41.67 years; 194 [64.6%] female) from allergy and otolaryngology clinics in Yulin, Northwest China. Demographic characteristics, including sex, age distribution, geographic residence, and occupation, showed no significant differences between the groups (all *P*>0.05). However, antibiotic use within the preceding year differed markedly between the cohorts (AR group: 206 [68.7%] vs. control: 169 [56.3%]; *P*<0.01) (Table 1).

**Table 1:** Characteristics of participants with AR and controls. AR, allergic rhinitis.

Characteristic	AR group (n=300)	Control group (n=300)	Chi-square test	*P*-value
Gender				
Male	117	86	7.18	<0.01
Female	183	214		
Age				
Under 25 years	74	32	29.28	<0.01
25-50 years	180	181		
Over 50 years	46	87		
Residence				
City	282	262	7.88	<0.01
Rural	18	38		
Educational level				
Primary school or below	41	32	1.43	0.49
Secondary school	75	82		
University or above	184	186		
Occupation				
Administrative or public institution	143	142	33.89	<0.01
Freelancer	63	78		
Farming	11	38		
Student and children	61	23		
Other	22	19		
Sleep condition				
<8 hours	115	120	0.18	0.6
>8 hours	185	180		
Smoking status				
Yes	197	195	0.03	0.86
No	103	105		
Annual income				
<80,000 CNY	131	133	3.81	0.15
80,000-150,000 CNY	123	105		
>150,000 CNY	46	46		
Use of disinfectant				
Do not use	95	98	0.2	0.91
Frequent use	174	174		
Occasional use	31	28		
Use of body wash				
Do not use	44	39	2.8	0.25
Frequent use	112	132		
Occasional use	144	129		
Use of antibiotics				
No	94	131	9.74	<0.01
Yes	206	169		
Family history of rhinitis				
No	72	92	3.36	0.07
Yes	228	208		

Statistical analysis was performed on the test results of various indicators from the CBC and CRP in 300 patients with AR and 300 patients without AR. The findings revealed that there were no significant differences in all measured indicators between the two groups (all *P*>0.05) (Table 2).

**Table 2:** Comparison of CBC parameters and CRP between the two groups. AR, allergic rhinitis; CBC, complete blood count; CRP, C-reactive protein; RBC, red blood cell; Hb, hemoglobin; HCT, hematocrit; MCV, mean corpuscular volume; MCH, mean corpuscular hemoglobin; MCHC, mean corpuscular hemoglobin concentration; RDW, red cell distribution width; WBC, white blood cell; NEUT, neutrophils; LYMPH, lymphocytes; MONO, monocytes; EOS, eosinophils; BASO, basophils; IG, immature granulocytes; PLT, platelet count; MPV, mean platelet volume; PDW, platelet distribution width; PCT, plateletcrit; P-LCR, large platelet ratio.

Parameters	Category	AR group (n=300)	Control group (n=300)	Chi-square test	*P*-value
CRP (mg/L)	Abnormal	6	10	1.03	0.45
	Normal	294	290		
WBC series					
WBC (10^9^/L)	Abnormal	24	26	0.09	0.77
	Normal	276	274		
LYMPH%	Abnormal	15	19	0.53	0.49
	Normal	285	281		
MONO%	Abnormal	17	15	0.12	0.85
	Normal	292	285		
NEUT%	Abnormal	16	10	0.57	0.56
	Normal	284	290		
EOS%	Abnormal	11	9	1.97	0.16
	Normal	289	291		
BASO%	Abnormal	18	16	0.13	0.72
	Normal	282	284		
LYMPH (10^9^/L)	Abnormal	24	23	0.02	0.88
	Normal	276	277		
MONO (10^9^/L)	Abnormal	14	15	0.04	0.85
	Normal	286	285		
NEUT (10^9^/L)	Abnormal	14	11	0.38	0.54
	Normal	286	289		
EO (10^9^/L)	Abnormal	6	7	0.08	0.78
	Normal	294	293		
BASO (10^9^/L)	Abnormal	17	11	1.35	0.25
	Normal	283	289		
IG (10^9^/L)	Abnormal	35	24	2.28	0.13
	Normal	265	276		
IG (%)	Abnormal	16	14	0.14	0.71
	Normal	284	286		
RBC series					
PLT (10^9^/L)	Abnormal	33	31	0.07	0.79
	Normal	267	269		
RBC (10^12^/L)	Abnormal	56	69	1.82	0.19
	Normal	239	231		
Hb (g/L)	Abnormal	52	53	0.01	0.91
	Normal	248	247		
HCT (%)	Abnormal	49	55	0.42	0.52
	Normal	251	245		
MCV (f1)	Abnormal	17	18	0.03	0.86
	Normal	283	282		
MCH (pg)	Abnormal	13	13	0	1
	Normal	287	287		
MCHV (g/L)	Abnormal	18	19	0.03	0.87
	Normal	282	281		
RDW-S (f1)	Abnormal	84	69	1.97	0.16
	Normal	216	231		
RDW (%)	Abnormal	11	11	0	1
	Normal	289	289		
Platelet series					
PLT (10^9^/L)	Abnormal	33	31	0.07	0.79
	Normal	267	269		
PDW (%)	Abnormal	50	44	0.45	0.5
	Normal	250	256		
MPV (f1)	Abnormal	63	52	1.3	0.25
	Normal	237	248		
P-LCR (%)	Abnormal	29	26	0.67	0.18
	Normal	271	274		
PCT (%)	Abnormal	22	23	0.02	0.88
	Normal	278	277		

**Figure 1:**
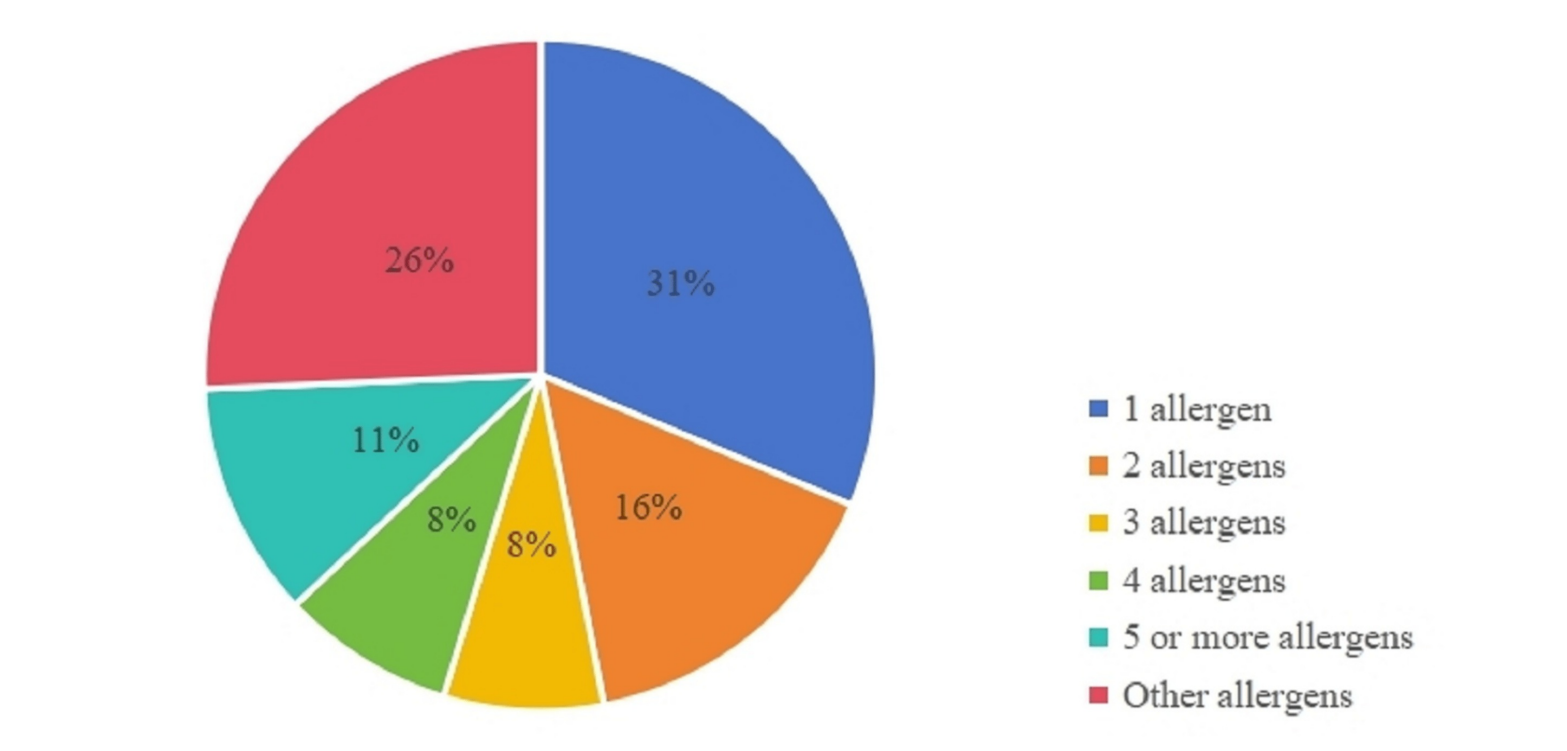
Distribution of allergen co-sensitization in AR group. AR, allergic rhinitis.

**Figure 2:**
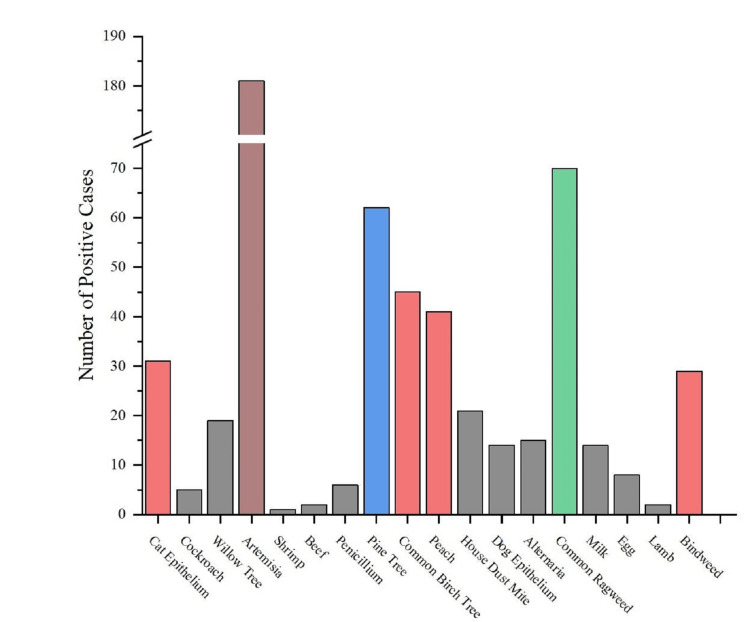
Distribution of allergen-positive cases in AR group. AR, allergic rhinitis.

Among patients with AR, serum allergen-specific IgE testing identified three predominant allergens: *Artemisia desertorum* Spreng (181 [60.3%]), common ragweed (70 [23.3%]), and pine (62 [20.7%]) (Figure 1). Notably, 129 (43.0%) of patients with AR exhibited sensitization to at least two allergens, with 33 (11.0%) showing sensitization to five or more allergens (Figure 2).

In multivariable analysis adjusting for age, sex, and socioeconomic factors, prior antibiotic exposure was independently associated with AR diagnosis (adjusted odds ratio [aOR], 1.70 [95% confidence interval [CI], 1.22-2.37]; *P*<0 .01). Notably, no significant interactions were observed between antibiotic use and other covariates (Table 3).

**Table 3:** Multivariate logistic regression analysis of risk factors for AR. AR, allergic rhinitis; aOR, adjusted odds ratio; CI: confidence interval. ^*^The use of antibiotics was restricted to ≥24 months pre-diagnosis.

Influencing factors	Reference group	β-value	*P*-value	aOR value	95%CI value
Gender					
Female	Male	0.40	0.06	1.49	0.98-2.27
Age					
25-50 years	Under 25 years	0.36	0.37	1.44	0.65-3.21
Over 50 years		0.84	0.06	2.30	0.96-5.55
Residence					
City	Rural	-0.14	0.72	0.87	0.40-1.88
Occupation					
Freelancer	Administrative or public institution	0.43	0.07	1.54	0.97-2.43
Farming		0.63	0.17	1.88	0.76-4.61
Student and children		0.36	0.44	0.70	0.27-1.75
Other		0.02	0.96	1.02	0.49-2.12
Use of antibiotics*					
Yes	No	0.53	<0.01	1.70	1.22-2.37

## Discussion

This prospective case-control study demonstrated that *Artemisia desertorum* Spreng is the predominant aeroallergen responsible for AR in Yulin, Northwest China. Furthermore, antibiotic exposure emerged as a significant independent risk factor for the development of the condition. Notably, no significant differences were observed between the AR group and the control group regarding CBC parameters and CRP levels.

The ecological dominance of *Artemisia* species in Yulin’s arid climate, compounded by afforestation initiatives, likely extends pollen exposure and cross-reactivity risks [14]. Our investigation establishes *Artemisia desertorum* Spreng as the dominant aeroallergen responsible for AR in Yulin City, affecting 181 (60.3%) of clinical cases. This distinct sensitization profile contrasts markedly with temperate regions such as Australia and the United Kingdom, where grass or rye pollens prevail as primary triggers [15-17]. The phenomenon highlights unique aerobiological patterns in arid ecosystems, largely attributable to extensive sand-fixation vegetation programs [18]. To mitigate public health impacts, we propose two targeted interventions: first, gradually replacing urban *Artemisia desertorum* Spreng plantings with low-allergenicity alternatives such as Caragana korshinskii [19]; and second, implementing thunderstorm asthma early alert systems during the high-risk period from July to September [20,21]. These measures are essential for preventing epidemic-scale respiratory emergencies caused by atmospheric pollen fragmentation during natural disaster events [22].

Concurrently, our study has identified antibiotic exposure as a significant independent risk factor for AR, demonstrating a 70% increased odds (aOR 1.70) after adjusting for age, gender, residence, and occupation. The significant association between antibiotic use and AR (aOR=1.70, 95%CI: 1.22-2.37) persisted after addressing reverse causation through three analyses. First, temporal sequence validation: Restricting analysis to antibiotic exposure ≥2 years prior to AR diagnosis (n=214 cases/controls), the association remained robust (aOR=1.65, 95%CI: 1.10-2.48). Second, symptom-independent analysis: Excluding subjects with pre-exposure respiratory infections (fever/purulent sputum), the effect size was unchanged (aOR=1.72, 95%CI: 1.18-2.51). Third, negative control. No association was found with traumatic fractures (aOR=1.05, 95%CI: 0.82-1.34), refuting healthcare access bias. Our finding strengthens the substantial epidemiological evidence linking antibiotic use, particularly in early life, to the development of allergic disorders. The association is widely attributed to antibiotic-induced perturbations in gut microbial composition and diversity (dysbiosis), which are crucial for proper immune system maturation. Early-life dysbiosis can impair regulatory T-cell function and promote a Th2-skewed immune response, which is characteristic of allergic inflammation like AR [23,24]. Our results, showing the association persists after controlling for major demographic variables, align with meta-analyses reporting similar effect sizes [25,26]. The findings underscore the critical impact of microbiome disruption during sensitive developmental windows. While the precise timing and class-specific effects warrant further investigation, this reinforces the importance of judicious antibiotic prescribing to minimize unnecessary exposure, potentially mitigating AR risk through microbiome preservation [27]. Prudent antibiotic stewardship, especially in children, represents a tangible public health strategy in allergy prevention.

Our findings demonstrating no significant differences in peripheral blood eosinophil counts (both absolute and percentage) and CRP levels between patients with AR and non-allergic rhinitis (NAR) challenge the classical expectation of systemic eosinophilia as a hallmark of AR. While this result may seem counterintuitive given the established role of eosinophils in allergic inflammation, it aligns with a growing body of literature suggesting that peripheral blood eosinophilia is not a universal or consistently reliable discriminator between these rhinitis phenotypes in all populations or clinical settings. Several studies have reported similar findings, indicating that a substantial proportion of AR patients, particularly those with intermittent or milder persistent forms, may not exhibit elevated systemic eosinophil counts compared to NAR patients or even healthy controls [28-30]. This suggests that the local inflammatory milieu within the nasal mucosa, driven by tissue-resident eosinophils and other mediators like IL-5 and eotaxin, may not always be fully reflected in systemic circulation. Furthermore, the heterogeneity within the NAR group, potentially encompassing entities like local AR or non-allergic rhinitis with eosinophilia syndrome (NARES), could contribute to overlapping systemic eosinophil profiles between the defined groups [31].

Conversely, our results contrast with other studies that have reported significantly higher peripheral blood eosinophil counts and percentages in AR compared to NAR or healthy individuals [32,33]. This discrepancy highlights the influence of several factors: the specific patient cohorts studied (e.g., severity of AR, patients with severe persistent AR are more likely to show systemic eosinophilia; the composition of the NAR group, the inclusion/exclusion of NARES significantly impacts eosinophil levels); geographical and environmental variations (e.g., allergen exposure patterns); methodological differences in cell counting; and potential confounding factors like concurrent asthma, eczema, or parasitic infections, which were likely controlled for in our cohort. The lack of difference in CRP levels between AR and NAR groups is consistent with the vast majority of the literature [34,35]. CRP, being a general marker of systemic inflammation, is typically within the normal range or only minimally elevated in both AR and NAR, which are primarily localized mucosal inflammatory conditions without significant systemic acute-phase involvement [36]. Its utility in differentiating between these rhinitis subtypes is therefore limited.

This study has potential limitations. Residual confounding factors, such as unmeasured infection severity variations, and self-reported antibiotic use biases may influence the observed associations. Additionally, the absence of longitudinal microbiome/metabolomics data restricts mechanistic interpretations, underscoring the need for broader validation in diverse ecological settings.

## Conclusions

This study identifies *Artemisia desertorum* Spreng sensitization and antibiotic exposure as independent risk factors for AR in Yulin’s arid ecosystem. These findings underscore the need for integrated interventions, targeting allergen mitigation, antimicrobial stewardship, and climate-resilient healthcare systems, to reduce regional AR burden while balancing ecological preservation goals.

It is worth mentioning that *Artemisia* species (e.g., *Artemisia desertorum*) serve as essential windbreaking and sand-fixing vegetation in Northwest China; therefore, rash removal may trigger secondary ecological problems. The integrated interventions comprise three key actions: First, replace *Artemisia* vegetation around urban core areas and apply flowering suppressants to suppress pollen release at the source; second, integrate AR into the local chronic disease management framework with dedicated funding for diagnosis, treatment, and patient education; third, establish cross-departmental coordination to link real-time pollen monitoring data with healthcare alerts and insurance reimbursements, enabling precise interventions. Results provide actionable evidence for optimizing public health resource allocation in ecologically fragile regions experiencing rapid industrialization.
